# Perception of Environmental Risks and Health Promotion Attitudes of French Perinatal Health Professionals

**DOI:** 10.3390/ijerph13121255

**Published:** 2016-12-18

**Authors:** Cécile Marie, Didier Lémery, Françoise Vendittelli, Marie-Pierre Sauvant-Rochat

**Affiliations:** 1 EA 4681, PEPRADE, Clermont Université, Université d’Auvergne, 28 Place Henri-Dunant BP 38, 63001 Clermont-Ferrand, France; dlemery@chu-clermontferrand.fr (D.L.); fvendittelli@chu-clermontferrand.fr (F.V.); m-pierre.sauvant-rochat@udamail.fr (M.-P.S.-R.); 2 Pôle Santé Publique, Centre Hospitalier Universitaire de Clermont-Ferrand, 58 Rue Montalembert, 63003 Clermont-Ferrand CEDEX 1, France; 3 Pôle Gynécologie-obstétrique, Centre Hospitalier Universitaire de Clermont-Ferrand, 58 Rue Montalembert, 63003 Clermont-Ferrand CEDEX 1, France; 4 Association des Utilisateurs de Dossiers informatisés en Pédiatrie, Obstétrique et Gynécologie (AUDIPOG), RTH Laennec Medical University, 7 rue Guillaume Paradin, 69372 Lyon CEDEX 08, France; 5 Département Santé Publique et Environnement, Faculté de Pharmacie, Clermont Université, Université d’Auvergne, 28 place Henri-Dunant BP 38, 63001 Clermont-Ferrand, France

**Keywords:** environmental chemicals, reproductive health, environmental health, perinatal health professionals, preventive attitude, risk perception

## Abstract

The exposure of pregnant women to environmental contaminants is a subject of international concern. However, the risk perception of these contaminants by health professionals (HP) has not been extensively investigated. The main objective of the PERI–HELPE study (Perception of Risk–HEaLth Professionals & Environment Study) was to assess the risk perception of environmental exposure of pregnant women by perinatal HPs. The secondary objectives were to describe the preventive attitudes of perinatal HPs concerning chemicals exposure of pregnant women and to identify the barriers to preventive attitude. A cross-sectional study was performed in 2015 in France. One hundred eighty-nine HPs (obstetricians, midwives, and general practitioners) replied to an online self-administered questionnaire (participation rate: 11%). Carbon monoxide, pesticides and lead were the contaminants most frequently perceived as a high risk for pregnant women. A minority of HPs asked women about their chemical exposure and advised them to reduce exposure. The lack of information, training and scientific evidence in environmental health were the main difficulties declared by the HPs to advise pregnant women. Despite the low response rate, our findings provide important information to encourage French health authorities to take into account the difficulties encountered by HPs and set up appropriate training programs in Environmental health.

## 1. Introduction

The exposure of pregnant women to environmental contaminants, such as pesticides, phthalates, bisphenol A, perfluorinated compounds, heavy metals and air pollutants, is currently a subject of international concern [1]. There is growing evidence about adverse obstetrical outcomes linked with in utero exposure to environmental contaminants such as spontaneous abortion, delayed fetal growth, premature birth, congenital malformations and impaired neural and cognitive development [2,3,4,5,6,7,8,9]. The International Federation of Gynecology and Obstetrics (FIGO) now recognizes these adverse health outcomes linked with prenatal exposure to environmental chemicals [1].

Accordingly, the FIGO has issued recommendations to perinatal health professionals (HP) and acknowledges their essential role in primary prevention for pregnant women [1]. The singular position of these clinicians has been emphasized by several American authors, who have provided guidance to clinicians to enable them to correctly inform women about how to avoid toxic exposure during pregnancy [10,11,12,13,14].

As pointed out by Grason and Misra [14], there are scant data about the knowledge and attitudes of perinatal HPs and about their perception of the risks associated with the exposure of pregnant women to environmental contaminants. A few studies on the perception or awareness of HPs of the hazards of plastic containers for food and drinks, air pollution and more generally the health risks of environmental contaminants showed the lack of professional knowledge about these issues [15,16,17]. To date, few studies have specifically dealt with risks related to exposure during pregnancy. In 2014, US obstetricians acknowledged their role in the prevention of prenatal environmental exposure but less than 20% reported routinely asking about environmental exposures, because they felt insufficiently informed [18]. However, these authors did not study the perception of risk related to different environmental factors or the provision of preventive advice that could help to decrease chemical exposure. Therefore, it is reasonable to enquire whether French perinatal HPs are properly aware of the risks entailed by environmental exposure during pregnancy and of the need to give preventive advice to pregnant women.

The main objective of the PERI-HELPE study (Perception of RIsk—HEaLth Professionals & Environment Study) was to assess the risk perception of perinatal HPs about exposure of pregnant women to environmental contaminants. The secondary objectives were to describe the health promotion attitudes of perinatal HPs towards chemical exposure of pregnant women and to identify the factors that might hinder preventive behavior.

## 2. Materials and Methods

### 2.1. Subjects

The study population was made up of perinatal HPs including senior or resident gynecologist-obstetricians (GO), general practitioners (GP) and senior or student midwives (MW). The study was conducted in Auvergne, a region in central France. In 2014, the region had 1,360,637 inhabitants and 13,430 births. To be eligible for inclusion, the HPs had to have pregnant women among their patients, be practicing in Auvergne and to agree to answer the questionnaire. In all, 194 perinatal HPs were included in the study.

In accordance with French human research law, the study was exempt from Institutional review approval because our database included no nominative data and the survey was not an interventional research study.

### 2.2. Study Design

**Figure 1:**
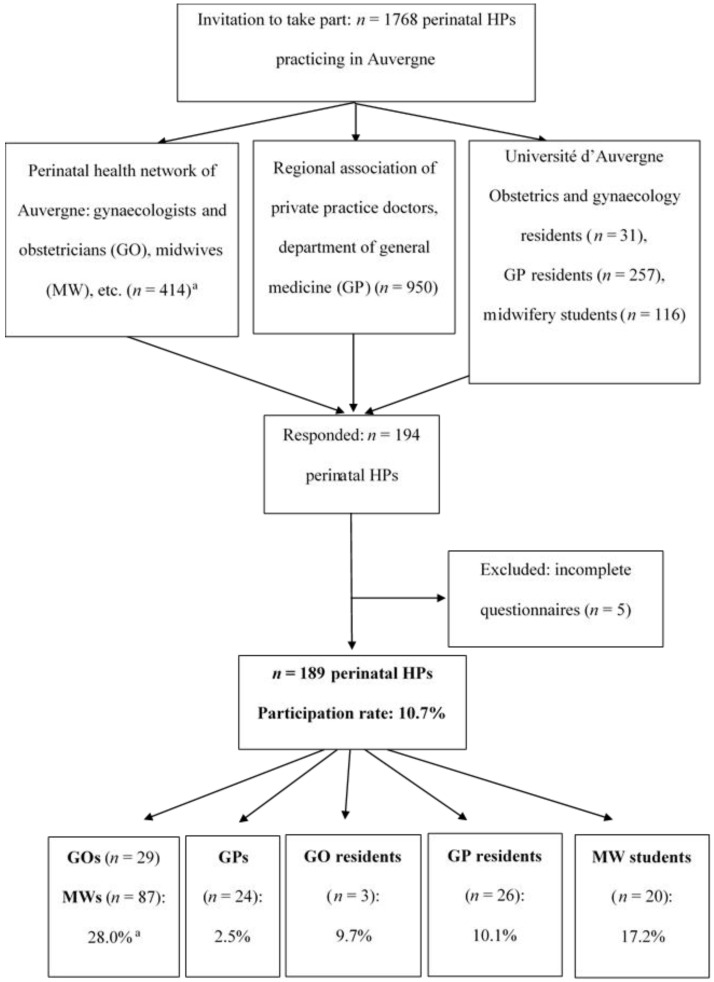
Participation rate of perinatal HPs in the PERI-HELPE study. GO, gynecologist-obstetricians; GP, general practitioners; HP, health professionals; MW, midwives. ^a^ The participation rate could not be calculated separately for GOs and MWs because the total number of each category of professionals in the network was not known.

A cross-sectional study was performed between 1 July and 31 December 2015. Before the study, the perinatal HPs were informed of the aim and modalities of the survey (completion of an online questionnaire). An invitation to take part was then sent by e-mail to 1768 perinatal HPs practicing in Auvergne, by various networks of professionals (Perinatal health network of Auvergne (RSPA), Regional association of private practice doctors, and the Department of general medicine and School of midwifery of the Université d’Auvergne). Three e-mail follow-ups were sent during the study period. Incomplete questionnaires were excluded (*n* = 5). The survey participation rate was 11% (189/1768) (Figure 1).

### 2.3. Data Collection

Data were collected by a standardized, anonymous and self-administered questionnaire. The design and online posting of the questionnaire were performed with Modalisa^®^ software, version 8.0 (Kynos, Paris, France, 2015). The questionnaire consisted predominantly of closed questions, with nine open questions. The questions concerned the socio-demographic and professional characteristics of the perinatal HPs, their knowledge and risk perception of various environmental contaminants, the identification of sources of environmental exposure, the preventive advice given to pregnant women, the sources of information about environmental health and the difficulties met with.

### 2.4. Assessment of Perception of the Risk to the in Utero Exposure of 19 Environmental Factors

The perinatal HPs replied to the question “Do you think that exposure to … presents a risk for the health of pregnant women and their unborn baby?” with a 5-point Likert scale (“don’t know”, “very low risk”, “rather low risk”, “high risk”, and “very high risk”). The environmental factors were based on a previous study on risk perception of environmental risks of French GPs [16]: pesticides, parabens, phthalates, bisphenol A, use of consumer products (household, do-it-yourself (DIY) and garden products), use of personal care products, food risk, outdoor air pollution, indoor air pollution, soil pollution, tap water quality, carbon monoxide, lead, radon, legionella, asbestos, waste incinerator, electromagnetic waves and noise.

### 2.5. Assessment of the Extent of Knowledge about Environmental Factors

The perinatal HPs self-assessed their ability to reply correctly to pregnant women’s enquiries about environmental factors with a 4-point Likert scale (“absolutely sure”, “fairly sure”, “fairly unsure”, and “totally unsure”).

### 2.6. Assessment of Preventive Attitudes towards Chemical Exposure of Pregnant Women

The health promotion attitudes of perinatal HPs were assessed by different items: (i) spontaneous questioning of pregnant women on their use of consumer products (household, DIY and gardening products) and personal care products (PCPs); (ii) advice about eating habits (avoid pre-packaged products, cling film for preserving food and micro-waving dishes in a plastic container, etc.) and the use of PCPs and consumer products during pregnancy (increase, decrease, choose less harmful products and/or take personal protection measures); and (iii) consider an environmental cause of an obstetric disorder. Provision of advice specifically related to pregnancy was also assessed: promotion of a healthy and balanced diet, prevention of toxoplasmosis (cook food thoroughly, and wash fruits and vegetables); encouragement to stop smoking, drinking alcohol and taking other drugs; and identifying hazards in the workplace. In France, these advices are set out in guidelines drawn up by the National Authority for Health [19].

### 2.7. Statistical Analysis

The qualitative variables were compared in the three subgroups of perinatal HPs (GP, GO and MW) with Pearson’s Chi-square or Fisher’s exact test, as appropriate. Significance was defined as *p* < 0.05. Statistical analyses were performed with R statistical software, version 2.15.2 (R Development Core Team, Vienna, Austria, 2012).

## 3. Results

More than half of the 189 perinatal HPs were MWs (56.6%), 26.5% were GOs and 16.9% GPs. The HPs were predominantly female (87.2%) with a mean age of 37.7 ± 12.4 years. The other sociodemographic and professional characteristics are given in Table 1.

### 3.1. Risk Perception of Exposure to Environmental Factors for Pregnant Women and Their Unborn Baby

**Figure 2:**
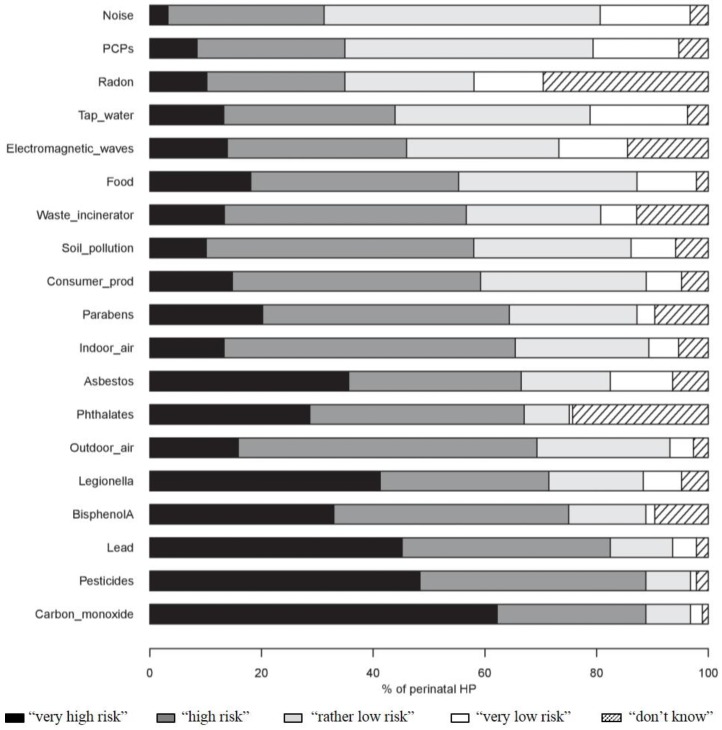
Perception of perinatal HPs of the risk of pregnant women and their unborn baby related to exposure to 19 environmental factors (*n* = 189). Consumer_prod, consumer products (corresponds to household, do-it-yourself (DIY) and gardening products); HP, health professionals; PCPs, personal care products.

More than 80% of the HPs considered that exposure to carbon monoxide, pesticides and lead was a “very high” or “high” risk for the health of pregnant women and their unborn baby. In contrast, more than 60% perceived exposure to noise, the use of PCPs and radon as being a “rather low” or “very low” risk. Questions about radon and phthalates were those most commonly answered by “don’t know” (respectively, 29.3% and 24.1%) (Figure 2). In most cases, the MWs were more likely than the other HPs to perceive contaminants as being of a “very high” or “high” risk (Table 2).

### 3.2. Enquiries to HPs by Pregnant Women about Environmental Issues

**Figure 3:**
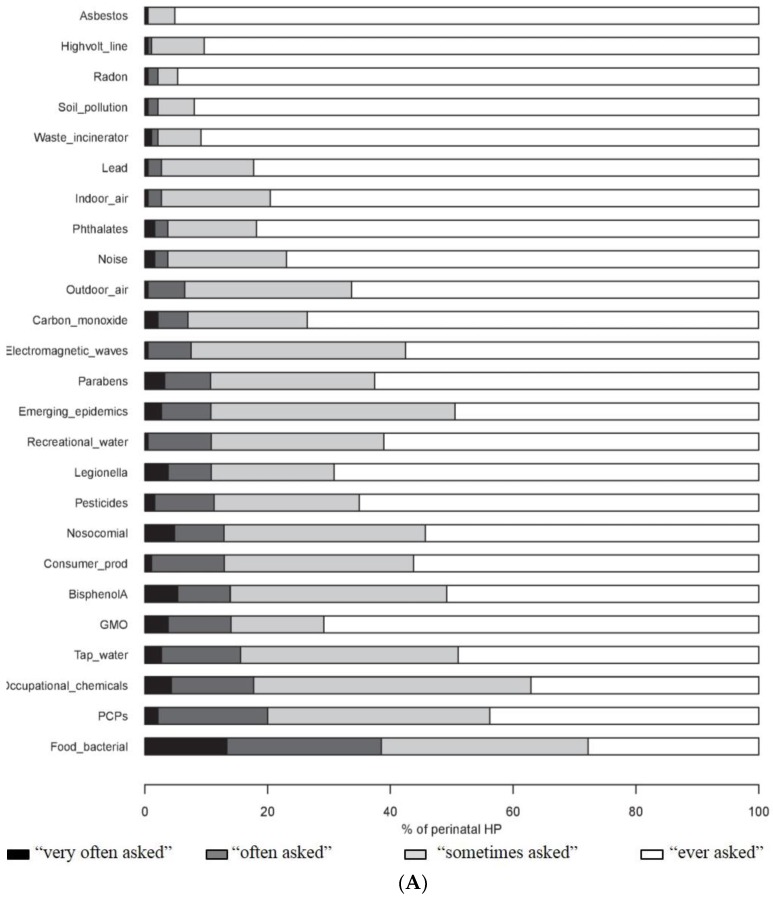

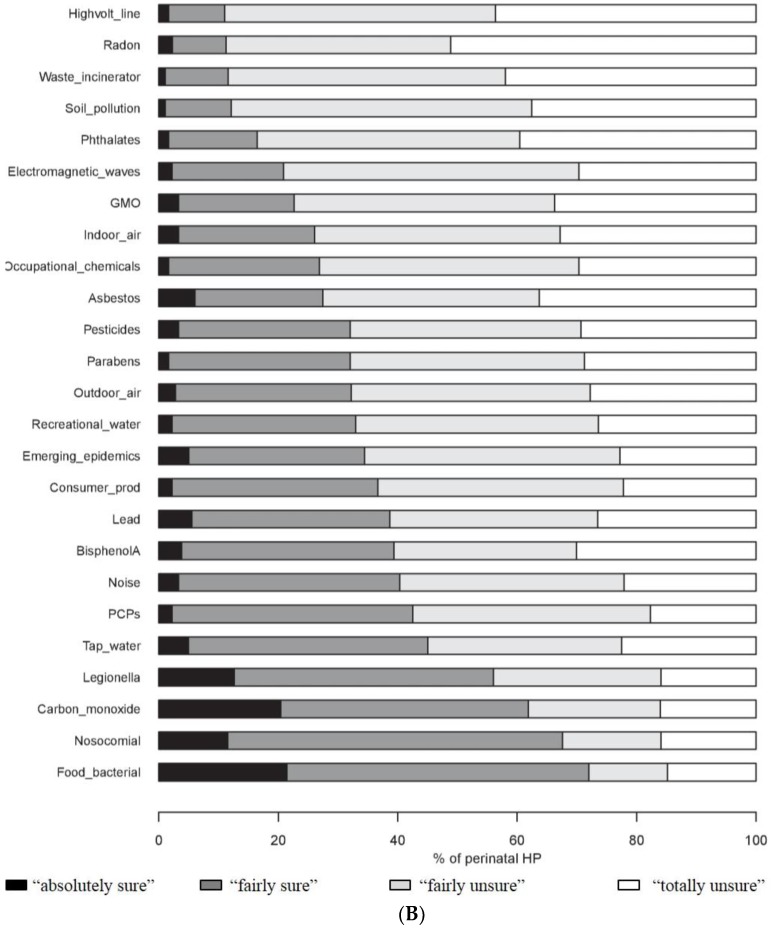
Enquiries made to perinatal HPs by pregnant women about environmental issues (**A**); and ability of perinatal HPs to provide appropriate answers (**B**). Consumer_prod, consumer products (corresponds to household, DIY and gardening products); GMO, genetically modified organism; HP, health professionals; PCPs, personal care products.

The HPs said they were “very often” or “often” asked by pregnant women about environmental hazards, in particular about risks related to contaminated food (38.5%), the use of PCPs (20.0%) and professional chemical exposure (17.7%). Asbestos, high-voltage power lines, radon, pollution of the soil and waste incinerators were the least commonly cited subjects of concern (Figure 3A). The MWs were more often questioned than the GPs and GOs about bisphenol A, parabens and the quality of tap water (*p* ≤ 0.05) (Supplementary Materials Table S1).

More than half of the HPs considered that they were “absolutely sure” or “fairly sure” of the answers they gave to enquiries of pregnant women about the risks of bacterial contamination of food, nosocomial infections, exposure to carbon monoxide and exposure to legionella. The environmental issues that most HPs (more than 80%) felt unable to address properly (“fairly unsure”, and “totally unsure”) were high-voltage power lines, radon, waste incinerators, pollution of the soil and phthalates (Figure 3B). Compared to the other HPs, the GOs stated more often that they were able to answer queries about new epidemics (*p* < 0.001) and GPs about indoor and outdoor air pollution and risks related to pesticides (*p* < 0.05) (Supplementary Materials Table S2).

### 3.3. Attitude of Perinatal HPs about Chemical Exposure of Pregnant Women

**Figure 4:**
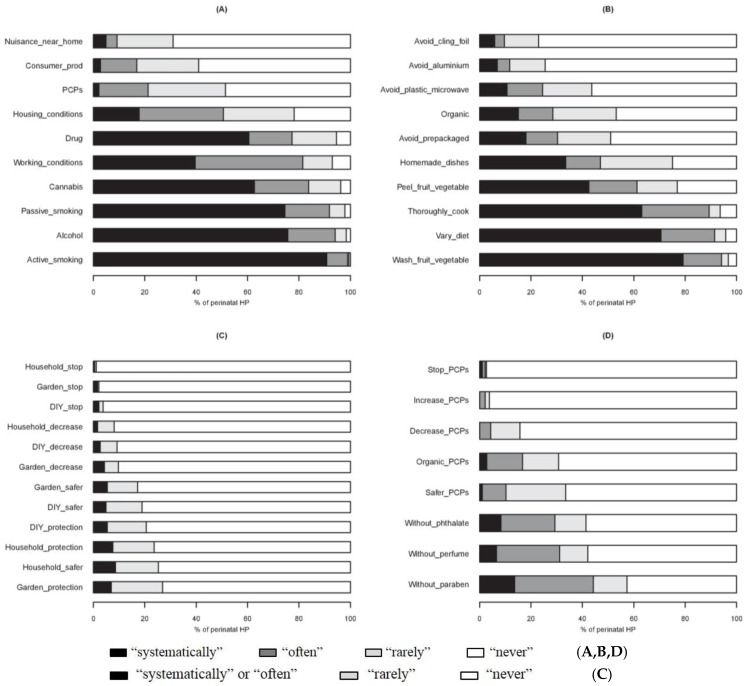
Attitude of perinatal HPs towards exposure of pregnant women to chemicals: spontaneous questioning about exposure factors (**A**); dietary advice (**B**); advice on the use of consumer products (**C**); and personal care products (**D**). Avoid_plastic_microwave, avoid micro-waving in plastic dishes; Consumer_prod, consumer products (corresponds to household, DIY and gardening products); HP, health professionals; PCPs, personal care products.

More than 90% of the HPs “systematically” or “often” questioned pregnant women about smoking (active and passive) and the consumption of alcohol. In contrast, few HPs asked questions “systematically” or “often” about environmental pollution in the vicinity of the home (9.2%), the use of consumer products (16.9%) or PCPs (21.3%) (Figure 4A). Questions about cannabis and other drugs were less often asked by the GPs (67.4% and 61.2%, respectively) than the other HPs (more than 80%) (*p* < 0.01).

More than 80% of the HPs advised pregnant women “systematically” or “often” to wash fruit and vegetables, to vary their diet and to cook food thoroughly. In contrast, less than 12% advised “systematically” or “often” not to use plastic kitchen cling film or aluminum foil for preserving food (Figure 4B). MWs tended to encourage the buying of organic food more often than the other HPs (*p* = 0.06) (Supplementary Materials Table S3).

Overall, HPs did not advise the women to change their use of consumer products during pregnancy. The wearing of individual protection and/or the use of less harmful products was advised “systematically” or “often” by less than 10% of the HPs (Figure 4C).

Very few HPs (4%) advised “systematically” or “often” to decrease the use of PCPs. Forty-four percent recommended products without paraben and 29% products without phthalates (Figure 4D). The MWs recommended significantly more often the use of PCPs not containing chemical products (without parabens, phthalates and synthetic fragrances and with an organic label) (Supplementary Materials Table S3).

**Figure 5:**
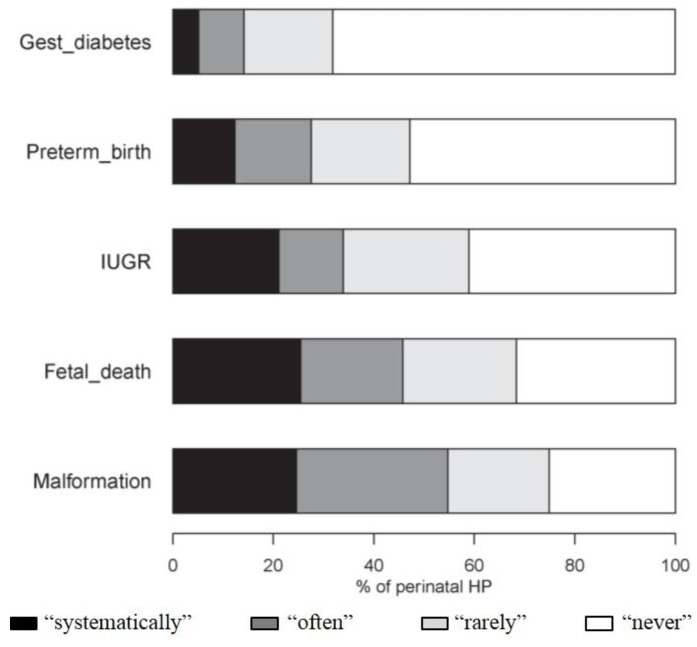
Investigation for exposure to environmental contaminants (except tobacco, alcohol and drugs) in the event of obstetric disorders. Gest_diabetes, gestational diabetes; HP, health professionals; IUGR, intrauterine growth restriction.

The HPs investigated “systematically” or “often” for exposure to environmental contaminants, particularly in the event of malformations (54.7%) and fetal death in utero (45.8%) (Figure 5). The GOs were significantly more likely to initiate such an investigation for both disorders (Supplementary Materials Table S3).

### 3.4. Factors Limiting the Health Promotion Attitudes of Perinatal HPs in Environmental Health

Most HPs felt “poorly” or “very poorly” informed about environmental contaminants (79.6%) and their effects on health (81.6%). Only 5.8% had received specific training in environmental health. Their main sources of information were the media (81.7%) and scientific articles (58.1%) (Table 3). Overall, the HPs considered that the information in scientific articles and that made available by health authorities was reliable (98.9% and 88.3% respectively). Only 27.8% expressed confidence in the media.

The lack of training, and hence of knowledge, in environmental health (57.6%), the lack of evidence-based medicine (54.5%) and the short duration of consultations (49.7%) were the most frequently difficulties cited by the HPs in properly informing pregnant women about risks related to environmental factors. Compared to the other HPs, the GOs were less likely to implicate environmental factors in obstetric disorders (Table 3).

## 4. Discussion

### 4.1. Perception of Environmental Risks by Perinatal HPs

To our knowledge, the perception of environmental risks of perinatal HPs has received scant attention. In this PERI-HELPE study, the perception of risks related to the exposure of pregnant women varied widely according to different environmental factors. The proportions of perinatal HPs perceiving a risk as being “very high” or “high” ranged from 32% (for noise) to 91% (for pesticides).

The main topics debated by the FIGO (pesticides, plasticizers, indoor air pollution, lead) [1] were often perceived as carrying a high environmental risk. The factors most commonly considered as being high risk (pesticides, carbon monoxide, air pollution, asbestos) were generally the same as in other recent studies in France involving GPs [16,20].

In the PERI-HELPE study, exposure to other products given less media coverage, such as PCPs, food and consumer products, was perceived as not being so great a hazard. However, it is acknowledged that certain eating habits, like the consumption of pre-packaged food and the use of kitchen cling film, and the use of PCPs and consumer products are associated with higher levels of exposure to endocrine disruptors such as benzophenone-3, bisphenol A, parabens, triclosan and phthalates [21,22,23,24,25,26]. In a French study involving GPs, food risks and those related to the use of chemical substances were among those the most frequently mentioned [16].

Other contaminants (for example, waste incinerators, and radon in homes) are also seen as carrying a low risk, both in our study and in studies involving senior managers or GPs [16,20,27]. However, it is difficult to compare our results with those of these three studies since they dealt with the perception of risk in general and not specifically with exposure of pregnant women.

### 4.2. Health Promotion Attitudes of Perinatal HPs

In France, the National Authority for Health has issued recommendations to HPs regarding information and preventive advice to be given to pregnant women [19]. The recommendations concern in particular risks related to the workplace, food infections, smoking, alcohol consumption, drugs use and lead poisoning. Our study shows that these recommendations are largely followed by perinatal HPs. For example, more than 90% of the HPs questioned pregnant women about smoking and alcohol consumption, and more than 80% gave advice about washing fruits and vegetables, varying diet and thoroughly cooking food (to prevent against toxoplasmosis); these attitudes were consistent with other studies [18,28,29].

In contrast, in our studied sample of perinatal HPs, giving advice to pregnant women about chemical exposure does not yet form part of the habits. Less than 12% of them “systematically” or “often” advised pregnant women to avoid using cling film or aluminum foil for preserving food, or to decrease their use of PCPs. These results are surprising. Respondents to the survey may be HPs motivated for environmental health prevention, and thus better able to provide ad hoc environmental health advice than non-respondents to the survey. However, these results are consistent with another study which showed that health personnel were inadequately informed about the possible risks of keeping food and drinks in plastic containers [15].

However, the important role that perinatal HPs can play in preventing pregnant women from chemical exposure, in particular to endocrine disruptors (such as pesticides, bisphenol A, phthalates) has been attested to in several American studies [10,11,12] and is acknowledged by the FIGO [1]. In Europe, the preventive role of perinatal HPs is less clearly defined. In some European countries, the health authorities inform women about chemical exposure during pregnancy [30,31], advise them to use eco-label household products, to decrease or avoid the use of cosmetics, regularly remove dust at home, and wash new kitchen utensils before use. These messages are directly addressed to pregnant women but before they are understood and adopted they may be called into question. Perinatal HPs could explain the message more fully and reinforce its impact, encouraging women to change their behavior and thereby to limit their exposure to environmental hazards.

### 4.3. Differences of Perception According to Health Professionals

The perception of risk related to the exposure of pregnant women to environmental hazards and the health promotion attitudes adopted varied according to the different categories of HPs in the PERI-HELPE study. Although the three subgroups of HPs had the same general feeling about their knowledge of environmental health, the MWs had a more acute perception of the risks of most of the contaminants than the other HPs. In addition, the MWs gave advice more often about decreasing chemical exposure, recommending organic food and chemical-free PCPs. The GOs asked more often about the consumption of cannabis and considered themselves more capable of giving reliable information about new epidemics. They also investigated more frequently for environmental factors in the event of fetal death in utero or of a malformation. The GPs were more likely to consider themselves capable of giving an informed answer to questions about air pollution and pesticides. These differences show that each type of HP seems to have a greater awareness of certain issues depending on acquired knowledge and skills.

These discrepancies of perception were also observed between specialist clinicians and community clinicians with regard to what role air pollution had in the occurrence and worsening of asthma symptoms in children [17]. Likewise, midwives and obstetricians questioned their patients more frequently about their consumption of cannabis and felt better informed about the risks involved than did GPs and gynecologists [32].

### 4.4. Need to Support the Involvement of Perinatal HPs in Environmental Health

In the PERI-HELPE study, the perinatal HPs were in general agreement about the main difficulties encountered in giving information and advice to pregnant women about exposure to environmental hazards. A lack of knowledge (cited by 58% of the HPs) and insufficient or contradictory scientific evidence (55%) have also been cited in other studies on the prevention of environmental risks in general [16] and during pregnancy [18,33]. Only 6% of the perinatal HPs had received training in environmental health.

Our study emphasizes the need to lend support to perinatal HPs to help them better inform pregnant women on how to prevent exposure to environmental hazards. Specialized training in environmental health (declared desirable by the participants in our study) would improve the level of knowledge of HPs. Educated providers could prompt conversations about environmental toxicants or act to reinforce women’s own knowledge. Studies on advice about smoking cessation have shown that when trained and fully informed HPs can be effective in changing pregnant women’s behavior [14,34,35]. Another difficulty mentioned in our study was the lack of time available during consultations. This drawback could be overcome by providing pregnant women with information in the form of brochures or leaflets, placing posters in waiting rooms and posting advice on the website of perinatal networks.

### 4.5. Limitations

Our study has limitations. It carries a risk of selection bias because the voluntary participants may have been more alert to the issues involved and more aware of environmental hazards. This selection bias would be limited, however, since the proportion of HPs who had received training in environmental health (5.8%) was not higher than that in a representative sample of French GPs (5%) [16] or that in a US study of obstetricians (6.7%) [18].

The major limitation of our study is its low participation rate (11%) which limits the extrapolation of the results observed in our low sample of perinatal HPs (*n* = 189) to the source population. Therefore, results should be taken with caution and further studies with better response rate and involving larger and more representative sample are needed to confirm our findings. Nonetheless, the overall response rate to our web-based questionnaire (11%) was similar to [18,36,37] or slightly lower [38,39] than that of other surveys in which HPs replied to an online questionnaire. The participation rate in our study varied from 2.5% (GPs) to 28.0% (GOs and MWs). Thus, lack of representativeness cannot be excluded, in particular for the GPs. It is possible that the GPs felt less concerned by our questionnaire than the other HPs such as the GOs and MWs. A French survey showed that among the HPs consulted during pregnancy, GPs were rarely involved (less than 5% vs. 67% for GOs) [28]. Compared to national data, GPs and GOs in our study were most often women and younger (however, we also included resident in contrast with national data). The proportion of GPs practicing in private practice and public health institution was consistent [40]. Moreover, the midwives—mostly health professionals included in our study—are representative for the population of French midwives in terms of sociodemographic and professional characteristics (gender, age and location of exercise, i.e., private practice or public health institution) [41], which improves the representativeness of the result.

Social desirability bias may have affected the HP’s self-reported responses, in particular to the questions about their attitudes. Although this bias is more frequent for sensitive areas such as illicit drug use and sexual behavior [42], it is possible that our participants were more likely to declare that they adopted preventive attitudes with pregnant women as evidence of responsible behavior and practice. However, this bias would be limited since our questionnaire was anonymous and online completed without an interview with an investigator. Moreover, the proportions of perinatal HPs who had preventive attitudes towards smoking, alcohol consumption and toxoplasmosis were similar to those in other studies [18,28,29]. Finally, it is difficult to compare our results concerning advice about avoiding chemical exposure and the perception of risk of a wide range of environmental factors because data are scare in the literature.

## 5. Conclusions

To our knowledge, the PERI-HELPE study is the first to describe the perception of risk of the exposure of pregnant women to environmental hazards by perinatal HPs. In our sample, most of the perinatal HPs taking part felt poorly informed about environmental health and did not give advice to pregnant women about limiting chemical exposure. However, the low participation rate limits the extrapolation of our results to the French perinatal HP population. Therefore, these results must be confirmed by a study carried out on a larger sample (with better response rate, and recruitment in several French regions). Despite this limitation, our findings suggest the need for increased education and training of perinatal HPs to enable them to have a better perception of risks related to chemical exposure during pregnancy. This study provides an important set of information to encourage French health authorities to take into account the difficulties identified and set up appropriate training schemes.

## Figures and Tables

**Table 1:** Sociodemographic and professional characteristics of perinatal health professionals (HPs).

	Total *n* = 189 (%)	GP ^a^ *n* = 50 (26.5%)	GO ^a^ *n* = 32 (16.9%)	MW ^a^ *n* = 107 (56.6%)	*p*-Value
**Sex**	*n* = 188	*n* = 50	*n* = 32	*n* = 106	
Female	164 (87.2)	37 (74.0)	21 (65.6)	106 (100.0)	<0.001
Male	24 (12.8)	13 (26.0)	11 (34.4)	0	
**Age (years)**	*n* = 188	*n* = 50	*n* = 32	*n* = 106	
<45 years	135 (71.8)	35 (70.0)	22 (68.8)	78 (73.6)	0.82
≥45 years	53 (28.2)	15 (30.0)	28 (31.2)	63 (26.4)	
**Parity**	*n* = 182	*n* = 47	*n* = 31	*n* = 104	
≥1	120 (65.9)	24 (51.1)	26 (83.9)	70 (67.3)	0.01
**Occupational status**	*n* = 189	*n* = 50	*n* = 32	*n* = 107	
Resident/mid-wife’s student	49 (25.9)	26 (52.0)	3 (9.4)	20 (18.7)	<0.001
Senior	140 (74.1)	24 (48.0)	29 (90.6)	87 (81.3)	
**Length of service**	*n* = 155	*n* = 39	*n* = 29	*n* = 87	
≤10 years	80 (51.6)	23 (59.0)	18 (62.1)	39 (44.8)	0.16
>10 years	75 (48.4)	16 (41.0)	11 (37.9)	48 (55.2)	
**Place of work** ^b^	*n* = 189	*n* = 50	*n* = 32	*n* = 107	
Private practice	63 (33.3)	38 (76.0)	5 (15.6)	20 (18.7)	<0.001
Private health institution	3 (1.6)	1 (2.0)	1 (3.1)	1 (0.9)	0.40
Public health institution	112 (59.3)	11 (22.0)	26 (81.3)	75 (70.1)	<0.001
**Contact with pregnant women**	*n* = 183	*n* = 48	*n* = 32	*n* = 103	
Consultation	52 (28.4)	36 (75.0)	8 (25.0)	8 (7.8)	<0.001
Hospital	17 (9.3)	1 (2.1)	2 (6.2)	14 (13.6)	
Both	91 (49.7)	11 (22.9)	22 (68.8)	58 (56.3)	
Others ^c^	23 (12.6)	0	0	23 (22.3)	

GO, gynecologist-obstetricians; GP, general practitioners; MW, midwives. ^a^ GP includes senior and residents GPs; GO includes senior and residents GOs; MW includes senior and students MWs; ^b^ Several possible replies for a given health professional; ^c^ Preparation for childbirth (*n* = 19) and/or pre- and postnatal home visit (*n* = 19).

**Table 2:** Perception of risk levels (“very high” or “high”) for the health of pregnant women and their unborn baby by perinatal health professionals (HPs).

	Number of Respondents ^a^	Number (%) of Perinatal HPs Perceiving a “Very high” or “High” Risk ^b^				
	*n* = 189	Total *n* (%)	GP ^c^ *n* (%)	GO ^c^ *n* (%)	MW ^c^ *n* (%)	*p*-Value
Pesticides	184	167 (90.8)	45 (93.8)	26 (81.3)	96 (92.3)	0.12
Carbon monoxide	186	167 (89.8)	41 (85.4)	26 (81.3)	100 (94.3)	0.05
Phthalates	140	124 (88.6)	28 (87.5)	17 (77.3)	79 (91.9)	0.15
Lead	184	155 (84.2)	39 (81.3)	25 (80.7)	91 (86.7)	0.58
Bisphenol A	170	141 (82.9)	34 (79.1)	20 (69.0)	87 (88.8)	0.03
Legionella	180	135 (75.0)	39 (79.6)	10 (33.3)	86 (85.2)	<0.001
Outdoor air pollution	184	131 (71.2)	31 (64.6)	19 (59.4)	82 (77.9)	0.06
Parabens	170	121 (71.2)	32 (71.1)	14 (50.0)	75 (77.3)	0.02
Asbestos	176	125 (71.0)	28 (57.1)	16 (57.1)	81 (81.8)	0.002
Indoor air pollution	178	123 (69.1)	32 (66.7)	14 (48.3)	77 (76.2)	0.02
Waste incinerator	163	106 (65.0)	29 (63.0)	15 (53.6)	62 (69.7)	0.28
Use of consumer products ^d^	180	112 (62.2)	29 (61.7)	13 (40.6)	70 (69.3)	0.01
Soil pollution	177	109 (61.6)	26 (54.2)	13 (44.8)	70 (70.0)	0.02
Food risk	184	104 (56.5)	24 (50.0)	9 (28.1)	71 (68.3)	<0.001
Electromagnetic waves ^e^	160	86 (53.8)	13 (34.2)	7 (25.0)	66 (70.2)	<0.001
Radon	131	65 (49.6)	16 (37.2)	3 (14.3)	46 (68.7)	<0.001
Tap water quality	182	83 (45.6)	16 (33.3)	8 (25.0)	59 (57.8)	<0.001
Use of PCPs	179	66 (36.9)	14 (39.8)	2 (6.7)	50 (49.0)	<0.001
Noise	180	58 (32.2)	16 (33.3)	7 (21.9)	35 (35.0)	0.38

GO, gynecologist-obstetricians; GP, general practitioners; MW, midwives; PCPs, personal care products. ^a^ For each environmental factor, the number of perinatal HPs having pronounced on the level of risk perceived (i.e., without those who replied “don’t know” and those who gave no reply); ^b^ Risk perceived as “very high” or “high” vs. “rather low” or “very low”; ^c^ GP includes senior and residents GPs; GO includes senior and residents GOs; MW includes senior and students MWs; ^d^ Consumer products corresponds to household, DIY and gardening products; ^e^ Electromagnetic waves: mobile phones, mobile phone masts, wifi, etc.

**Table 3:** Sources of information and difficulties encountered by perinatal health professionals (HPs) in advising pregnant women about environmental health (EH).

	Total *n* = 189 (%)	GP ^a^ *n* = 50 (%)	GO ^a^ *n* = 32 (%)	MW ^a^ *n* = 107 (%)	*p*-Value
***Declared level of EH knowledge***					
**Environmental contaminants**	*n* = 189	*n* = 50	*n* = 32	*n* = 107	
“Well informed” ^b^	38 (20.1)	11 (22.0)	3 (9.4)	24 (22.4)	0.39
“Poorly informed”	125 (66.1)	30 (60.0)	24 (75.0)	71 (66.4)	
“Very poorly informed”	26 (13.8)	9 (18.0)	5 (15.6)	12 (11.2)	
**Health outcomes**	*n* = 188	*n* = 49	*n* = 32	*n* = 107	
“Well informed” ^b^	34 (18.1)	12 (24.5)	6 (18.8)	16 (15.0)	0.57
“Poorly informed”	117 (62.2)	29 (57.1)	18 (56.3)	71 (66.4)	
“Very poorly informed”	37 (19.7)	9 (18.4)	8 (25.0)	20 (18.7)	
**Training in EH** (“Yes”) ^c^	*n* = 189	*n* = 50	*n* = 32	*n* = 107	
	11 (5.8)	2 (4.0)	1 (3.1)	8 (7.5)	0.36
**Desire training in EH** (“Yes”)	*n* = 186	*n* = 50	*n* = 32	*n* = 104	
	142 (76.3)	38 (76.0)	22 (68.8)	82 (78.9)	0.50
**Sources of information about EH**	*n* = 189	*n* = 50	*n* = 32	*n* = 107	
Media, Internet	154 (81.5)	35 (70.0)	25 (78.1)	94 (87.9)	0.02
Scientific article	109 (57.7)	31 (62.0)	17 (53.1)	61 (57.0)	0.71
Health agency or institution	40 (21.1)	15 (30.0)	2 (6.3)	23 (21.5)	0.04
Environmental association	37 (19.6)	11 (22.0)	5 (15.6)	21 (19.6)	0.78
Other health professionals	28 (14.8)	5 (10.0)	4 (12.5)	19 (17.8)	0.41
Other source	10 (5.3)	4 (8.0)	2 (6.3)	4 (3.7)	0.49
**Difficulties**	*n* = 189	*n* = 50	*n* = 32	*n* = 107	
Lack of knowledge	110 (58.2)	27 (54.0)	15 (46.9)	68 (63.6)	0.19
Insufficient/contradictory scientific evidence	103 (54.5)	28 (56.0)	20 (62.5)	55 (51.4)	0.53
Lack of time during consultation	95 (50.3)	23 (46.0)	15 (46.9)	57 (53.2)	0.64
Lack of interest of women	66 (34.9)	14 (28.0)	12 (37.5)	40 (37.4)	0.49
Low contribution of environmental factor to disorder	48 (25.4)	13 (26.0)	18 (56.3)	17 (15.9)	<0.001
Contact with women too late in pregnancy	38 (20.1)	5 (10.0)	5 (15.6)	28 (26.2)	0.05
Lack of interest	16 (8.5)	6 (12.0)	2 (6.3)	8 (7.5)	0.56

GO, gynecologist-obstetricians; GP, general practitioners; MW, midwives. ^a^ GP includes senior and residents GPs; GO includes senior and residents GOs; MW includes senior and students MWs; ^b^ “Very well informed” (*n* = 1) and “fairly well informed”; ^c^ Master’s degree (*n* = 1), university diploma (*n* = 2), other training experience (*n* = 7).

## Supplementary Materials

The following are available online at http://www.mdpi.com/1660-4601/13/12/1255/s1, Table S1: Enquiries to perinatal HPs by pregnant women about environmental issues, Table S2: Ability of perinatal HPs to provide appropriate answers to enquires of pregnant women, Table S3. Advice given to pregnant women by perinatal HPs.

## Abbreviations

FIGO	International Federation of Gynecology and Obstetrics
GO	gynaecologist-obstetricians
GP	general practitioner
HP	health professionals
MW	midwife
PCPs	personal care products
PERI-HELPE study	Perception of RIsk—HEaLth Professionals & Environment Study
