# Supplementary material for: Perception of Environmental Risks and Health Promotion Attitudes of French Perinatal Health Professionals
Source: Int J Environ Res Public Health. 2016 Dec 18;13(12):1255. doi: 10.3390/ijerph13121255 (PMC5201396; doi:10.3390/ijerph13121255)
Supplement: Supplementary file 1 [file ijerph-13-01255-s001.pdf]

International Journal of
Environmental Research and Public Health

MDPI

Supplementary Materials: Perception of Environmental Risks and Health Promotion Attitudes of French Perinatal Health Professionals

Cécile Marie, Didier Lémery, Françoise Vendittelli and Marie-Pierre Sauvant-Rochat

Table S1. Enquiries to perinatal HPs by pregnant women about environmental issues [a].

	Total n = 189 (%)	GP [b] n = 50 (%)	GO [b] n = 32 (%)	MW [b] n = 107 (%)	p-Value
Asbestos	9/184 (4.9)	5 (10.2)	1 (3.1)	3 (2.9)	0.17
Bisphenol A	92/187 (49.2)	17 (34.7)	11 (34.4)	64 (60.4)	0.002
Noise	43/186 (23.1)	9 (18.4)	11 (34.4)	23 (21.9)	0.22
Occupational exposure to chemicals	117/186 (62.9)	25 (51.0)	23 (71.9)	69 (65.7)	0.11
Waste incinerators	17/186 (9.1)	6 (12.2)	2 (6.3)	9 (8.6)	0.63
Nosocomial infections	85/186 (45.7)	21 (42.9)	13 (40.6)	51 (48.6)	0.66
Legionella	57/185 (30.8)	18 (36.7)	5 (15.6)	34 (32.7)	0.11
Power lines	18/186 (9.7)	8 (16.3)	2 (6.3)	8 (7.6)	0.18
Carbon monoxide	49/185(26.5)	8 (16.3)	12 (37.5)	29 (27.9)	0.10
Emerging epidemics	94/186 (50.5)	20 (40.8)	16 (50.0)	58 (55.2)	0.25
Electromagnetic waves	79/186 (42.5)	22 (44.9)	10 (31.3)	47 (44.8)	0.37
Parabens	70/187 (37.4)	14 (28.6)	8 (25.0)	48 (45.3)	0.04
Lead	33/186 (17.7)	7 (14.3)	5 (15.6)	21 (20.0)	0.65
Phthalates	34/187 (18.2)	7 (14.3)	4 (12.5)	23 (21.7)	0.35
Outdoor air pollution	62/184 (33.7)	19 (39.6)	10 (31.3)	33 (31.7)	0.60
Indoor air pollution	38/186 (20.4)	13 (26.5)	3 (9.4)	22 (21.0)	0.17
Soil pollution	15/186 (8.1)	5 (10.2)	2 (6.3)	8 (7.6)	0.79
Tap water quality	95/186 (51.1)	23 (46.9)	11 (34.4)	61 (58.1)	0.05
Recreational water quality	72/185 (38.9)	21 (42.9)	10 (32.3)	41 (39.1)	0.64
Radon	10/186 (5.4)	5 (10.2)	1 (3.1)	4 (3.8)	0.22
Food risks—bacterial contamination	135/187 (72.2)	35 (71.4)	24 (75.0)	76 (71.7)	0.93
Food risks—GMO	54/185 (29.2)	16 (33.3)	7 (21.9)	31 (29.5)	0.54
Food risks—pesticides	65/186 (34.9)	20 (40.8)	8 (25.0)	37 (34.2)	0.34
Use of PCPs	104/185 (56.2)	21 (42.9)	18 (56.3)	65 (62.5)	0.07
Use of consumer products [c]	81/185 (43.8)	21 (42.9)	13 (40.6)	47 (45.2)	0.89

DIY, do-it-yourself; GMO, genetically modified organism; GO, gynaecologist-obstetricians; GP, general practitioners; HP, health professionals; MW, midwives; PCPs, personal care products. [a] Enquiries (“sometimes”, “often”, “very often” vs. “never”). [b] GP includes senior and residents GPs; GO includes senior and residents GOs; MW includes senior and students MWs. [c] Consumer products corresponds to household, DIY and gardening products.

Table S2. Ability of perinatal HPs to provide appropriate answers to enquires of pregnant women [a].

	Total n = 189 (%)	GP [b] n = 50 (%)	GO [b] n = 32 (%)	MW [b] n = 107 (%)	p-Value
Asbetos	50/182 (27.5)	18 (36.7)	11 (35.5)	21 (20.6)	0.06
Bisphenol A	72/183 (39.3)	15 (30.6)	13 (41.9)	44 (42.7)	0.34
Noise	73/181 (40.3)	22 (44.9)	13 (41.9)	39 (37.6)	0.68
Occupational exposure to chemicals	49/182 (26.9)	15 (30.6)	8 (25.8)	26 (25.5)	0.79
Waste incinerator	21/181 (11.6)	5 (10.2)	6 (19.4)	10 (9.9)	0.33
Nosocomial infections	123/182 (67.6)	35 (71.4)	19 (61.3)	69 (67.7)	0.64
Legionella	102/182 (56.0)	33 (67.4)	16 (51.6)	53 (52.0)	0.18
Power lines	20/181 (11.5)	8 (16.7)	4 (12.9)	8 (7.8)	0.26
Carbon monoxide	112/181 (61.9)	31 (64.6)	23 (74.2)	58 (56.9)	0.20
Emerging epidemics	62/180 (34.4)	19 (38.8)	18 (58.1)	25 (25.0)	0.002
Electromagnetic waves	38/182 (20.9)	12 (24.5)	6 (19.4)	20 (19.6)	0.77
Parabens	58/181 (32.0)	14 (28.6)	10 (32.3)	34 (33.7)	0.82
Lead	70/181 (38.7)	24 (49.0)	14 (45.2)	32 (31.7)	0.09

Table S2. *Cont.*

	Total n = 189 (%)	GP [b] n = 50 (%)	GO [b] n = 32 (%)	MW [b] n = 107 (%)	p-Value
Phthalates	30/182 (16.5)	8 (16.3)	4 (12.9)	18 (17.7)	0.82
Outdoor air pollution	58/180 (32.2)	22 (45.8)	6 (19.4)	30 (29.7)	0.03
Indoor air pollution	47/180 (26.1)	19 (38.8)	4 (13.3)	24 (23.8)	0.03
Soil pollution	22/181 (12.2)	9 (18.4)	3 (9.7)	10 (9.9)	0.30
Tap water quality	82/182 (45.1)	22 (44.9)	16 (51.6)	44 (43.1)	0.71
Recreational water quality	60/182 (32.6)	16 (32.7)	10 (32.3)	34 (33.3)	0.99
Radon	20/178 (11.2)	9 (18.0)	2 (6.4)	9 (9.0)	0.12
Food risks—bacterial contamination	131/182 (72.0)	35 (71.4)	21 (70.0)	75 (72.8)	0.95
Food risks—GMO	421181 (22.7)	14 (28.6)	8 (25.8)	19 (18.8)	0.37
Food risks—pesticides	58/181 (32.0)	22 (44.9)	6 (19.4)	30 (29.7)	0.04
Use of PCPs	77/181 (42.5)	21 (42.9)	12 (38.7)	44 (43.6)	0.89
Use of consumer products [c]	66/180 (36.7)	23 (47.9)	9 (29.0)	34 (33.7)	0.15

DIY, do-it-yourself; GMO, genetically modified organism; GO, gynaecologist-obstetricians; GP, general practitioners; MW, midwives; PCPs, personal care products. [a] Ability to answer correctly ("absolutely sure" or "fairly sure") to enquiries by pregnant women vs inability to answer correctly ("fairly unsure" or "totally unsure"). [b] GP includes senior and residents GPs; GO includes senior and residents GOs; MW includes senior and students MWs. [c] Consumer products corresponds to household, DIY and gardening products.

Table S3. Advice given to pregnant women by perinatal HPs.

	Total n = 189 (%)	GP n = 50 (%)	GO n = 32 (%)	MW n = 107 (%)	p-Value
	Advice about eating habits [a]				
Wash fruits/vegetables	176/187 (94.1)	46 (92.0)	29 (93.6)	101 (95.3)	0.62
Peel fruits/vegetables	114/186 (61.3)	36 (72.0)	21 (67.7)	57 (54.3)	0.08
Thoroughly cook food	167/187 (89.3)	45 (90.0)	26 (81.3)	96 (91.4)	0.26
Vary diet	171/187 (91.4)	43 (96.0)	27 (87.1)	101 (95.3)	0.1
Prefer homemade dishes	87/185 (47.0)	18 (36.7)	17 (54.8)	52 (49.5)	0.21
Avoid pre-packaged food	57/188 (30.3)	13 (26.0)	8 (25.3)	36 (34.0)	0.46
Organic food	53/186 (28.5)	13 (26.5)	4 (12.9)	36 (34.0)	0.06
Avoid micro-waving in plastic dishes	46/188 (24.5)	10 (20.0)	8 (25.0)	28 (26.4)	0.68
Avoid cling foil	18/187 (5.3)	4 (8.0)	4 (12.5)	10 (9.5)	0.80
Avoid aluminum	22/188 (11.7)	4 (8.0)	4 (12.5)	14 (13.2)	0.63
	**Reasons for advice on eating habits**				
Prevent chemical risk	53/189 (28.0)	14 (28.0)	8 (25.0)	31 (29.0)	0.91
Prevent infections	177/189 (93.7)	47 (94.0)	31 (96.9)	99 (92.5)	0.67
	Advice about use of PCPs				
**Modification of use** [a]					
Use more often	4/185 (2.2)	2 (4.0)	0	2 (1.9)	0. 63
Stop/decrease	12/185 (6.5)	2 (4.0)	3 (9.4)	7 (6.8)	0.60
Use less harmful products	19/185 (33.5)	3 (6.0)	1 (3.1)	15 (14.6)	0.12
**Types of PCPs advised** [a]					
Organic	30/179 (16.8)	5 (10.0)	1 (3.5)	24 (24.0)	0.01
Without synthetic fragrances	57/183 (31.1)	8 (16.0)	7 (24.1)	42 (40.4)	0.006
Without parabens	81/183 (44.3)	15 (30.0)	9 (31.0)	57 (54.8)	0.004
Without phthalates	53/181 (29.3)	11 (22.0)	5 (17.2)	37 (36.3)	0.06
	Advice about use of household products [a]				
Stop/decrease	4/186 (2.2)	1 (2.0)	1 (3.1)	2 (1.9)	0.81
Use less harmful products	16/186 (8.6)	7 (14.0)	1 (3.1)	8 (7.7)	0.22
Wear protection	14/186 (7.5)	7 (14.0)	1 (3.1)	6 (5.8)	0.14
	Advice about use of DIY products [a]				
Stop/decrease	9/185 (4.9)	3 (6.0)	2 (6.3)	4 (3.9)	0.71
Use less harmful products	9/185 (4.9)	4 (8.0)	1 (3.1)	4 (3.9)	0.58
Wear protection	10/185 (5.4)	4 (8.0)	1 (3.1)	5 (4.9)	0.66

Table S3. *Cont.*

	Total<br>*n* = 189 (%)	GP<br>*n* = 50 (%)	GO<br>*n* = 32 (%)	MW<br>*n* = 107 (%)	*p*-Value
Advice about use of gardening products [a]					
Stop/decrease	11/186 (5.9)	3 (6.0)	2 (6.3)	6 (5.8)	1
Use less harmful products	10/186 (5.4)	4 (8.0)	3 (9.4)	3 (2.9)	0.20
Wear protection	13/186 (7.0)	4 (8.0)	2 (6.3)	7 (6.7)	0.92
Reasons for advice about household, DIY and gardening products					
Prevention of preterm birth	25/73 (34.2)	6 (31.6)	5 (35.7)	14 (35.0)	0.96
Prevention of exposure to chemicals	60/73 (82.2)	16 (31.6)	10 (35.7)	34 (85.0)	0.50
Investigation for environmental exposure in the event of a disorder [a]					
Malformation	98/179 (54.7)	18 (37.5)	25 (78.1)	55 (55.6)	0.001
Fœtal death	81/177 (45.8)	16 (34.8)	20 (62.5)	45 (45.5)	0.05
Intrauterine growth restriction	61/180 (33.9)	17 (35.4)	14 (43.8)	30 (30.0)	0.38
Preterm birth	49/178 (27.5)	13 (28.3)	10 (31.3)	26 (26.0)	0.84
Gestational diabetes	25/176 (14.2)	7 (15.2)	3 (9.4)	15 (15.3)	0.69

DIY, do-it-yourself; GO, gynaecologist-obstetricians; GP, general practitioners; MW, midwives; PCPs, personal care products. [a] Advice given "systematically" or "often" vs. "rarely" or "never".

© 2016 by the authors; licensee MDPI, Basel, Switzerland. This article is an open access article distributed under the terms and conditions of the Creative Commons Attribution (CC-BY) license (http://creativecommons.org/licenses/by/4.0/).
